# Emerging role of complement system in the induction of neuroinflammation in adenylosuccinate lyase deficiency disorder

**DOI:** 10.1016/j.bbih.2025.101091

**Published:** 2025-08-19

**Authors:** Albert Frank Magnusen, Robert James Hopkin, Charles Vorhees, Elizabeth Wilson, Molly Moehlman, Barbara Hallinan, Craig Erickson, Melissa P. DelBello, Luca Marsili, Nicole G. Coufal, Manoj Kumar Pandey

**Affiliations:** a Division of Human Genetics, Cincinnati Children's Hospital Medical Center, Cincinnati, OHIO, USA; b Division of Neurology, Cincinnati Children's Hospital Medical Center, Cincinnati, OH, USA; c The Kelly O'Leary Center for Autism Spectrum Disorders, Cincinnati Children's Hospital Medical Center, Cincinnati, OH, USA; d Department of Psychiatry, College of Medicine, University of Cincinnati, Cincinnati, OH, USA; e James J. and Joan A. Gardner Center for Parkinson's Disease and Movement Disorders, University of Cincinnati, OH, USA; f Department of Pediatrics, University of California San Diego and Rady Children's Hospital, University of California, San Diego, USA; g Department of Pediatrics, College of Medicine, University of Cincinnati, Cincinnati, OH, USA

**Keywords:** Genetic defect, Rare disease, Neuroinflammation, Complement activation

## Abstract

Adenylosuccinate lyase deficiency disorder (ADSLDD) is an ultra-rare autosomal recessive metabolic condition that leads to severe neurological impairment, with an estimated global prevalence of approximately 0.00125 cases per 100,000 individuals. Clinically, ADSLDD presents in three distinct phenotypes: the fatal neonatal form, the childhood form, and the more slowly progressive form, each characterized by varying degrees of developmental and neurological dysfunction. The disorder is caused by pathogenic variants in the ADSL gene, leading to impaired enzymatic activity and the accumulation of toxic substrates particularly succinyladenosine (S-Ado) and succinylaminoimidazole carboxamide riboside (SAICAr).

The ratio of S-Ado to SAICAr in cerebrospinal fluid has been correlated with disease severity, where lower ratios are associated with more severe clinical outcomes. However, the precise mechanisms linking elevated SAICAr levels to neurological damage remain incompletely understood.

This review summarizes current insights into the metabolic dysfunction and immune activation observed in ADSLDD, with a focus on the role of SAICAr in promoting neuroinflammation. We highlight emerging hypotheses implicating activation of the alternative complement pathway as a key driver of inflammation, blood-brain barrier disruption, and progressive neurodegeneration.

By synthesizing recent findings, this review underscores the urgent need for mechanistic studies and therapeutic exploration, particularly targeting complement activation, as a promising strategy to mitigate inflammation and improve clinical outcomes in ADSLDD.

## 1 Introduction

Adenylosuccinate lyase deficiency disorder (ADSLDD) is an ultra-rare autosomal recessive inborn error of purine metabolism that results in a broad spectrum of neurological impairments, including developmental delay, epilepsy, intellectual disability, and autistic traits (Cutillo et al., 2024; Moro et al., 2023; Andelman-Gur et al., 2020). In general, a genetic rare disease is defined as a heritable condition affecting fewer than 1 in 2000 individuals in Europe or fewer than 200,000 individuals in the United States (Smith et al., 2022). ADSLDD is significantly rarer than this threshold, with an estimated global prevalence of approximately 0.00125 cases per 100,000 individuals, qualifying it as an ultra-rare disorder (Cutillo et al., 2024; Mastrogiorgio et al., 2021; Jurecka et al., 2015).

Although initially identified in European populations particularly in the Netherlands and Belgium, cases of ADSLDD have since been reported globally, including in Australia, China, the Czech Republic, Poland, the United Kingdom, France, Germany, Italy, Morocco, Norway, Portugal, Spain, Turkey, Qatar, Israel, and the United States (Mastrogiorgio et al., 2021; Jurecka et al., 2015; Cakmak Celik et al., 2021; Wang et al., 2022).

Clinically, ADSLDD manifests in three major phenotypes; the fatal neonatal form, the childhood form, and the more slowly progressive form. The fatal neonatal form, the most severe, presents in utero or at birth and is characterized by profound neurological dysfunction, microcephaly, hypotonia, intractable seizures, and early mortality. The childhood form (type I) typically begins later in infancy and includes significant psychomotor delays, hypotonia, epilepsy, and frequent autistic features. The slowly progressive form (type II) is milder, often with near-normal early development followed by later-onset neurodevelopmental regression, seizures, ataxia, and behavioral disturbances (Mastrogiorgio et al., 2021; Donti et al., 2016; Jurecka et al., 2014; Mouchegh et al., 2007).

At the molecular level, ADSLDD results from pathogenic mutations in the ADSL gene, a ∼23-kilobase region located on chromosome 22q13.1–13.2. This gene encodes adenylosuccinate lyase (ADSL; EC 4.3.2.2), a highly conserved enzyme critical for both de novo purine biosynthesis and the purine nucleotide cycle. ADSL catalyzes two essential non-redundant reactions: the conversion of succinylaminoimidazole carboxamide ribotide (SAICAR) to aminoimidazole carboxamide ribotide (AICAR) in the de novo purine synthesis pathway, and the transformation of succinyladenosine monophosphate (SAMP) to adenosine monophosphate (AMP) in the purine nucleotide cycle. Through these reactions, ADSL contributes to the maintenance of nucleotide pools necessary for DNA/RNA synthesis and cellular energy metabolism, defining its fundamental role in neurodevelopmental and metabolic homeostasis (Jurecka et al., 2015; Van Keuren et al., 1987; Fon et al., 1993).

To date, over 80 pathogenic variants have been identified in Adenylosuccinate lyase deficiency disorder, including missense variants (the most common), nonsense variants, splice-site mutations, small deletions or insertions, and compound heterozygous combinations (Ferreira, 2017). Missense mutations tend to be associated with residual enzyme activity and are often linked to milder phenotypes (type I or II). In contrast, nonsense mutations or mutations affecting splicing or protein folding often result in near-complete loss of function, correlating with the fatal neonatal form (Mastrogiorgio et al., 2021; Wang et al., 2022).

Structural studies of ADSL protein have shown that certain missense variants destabilize its tetrameric configuration or alter its catalytic domains, impairing its ability to interact with substrates (Ray et al., 2012). As a result of ADSL deficiency, metabolic intermediates accumulate, particularly the dephosphorylated substrates SAICA riboside (SAICAr) and succinyladenosine (S-Ado), which are readily detectable at elevated levels in plasma, cerebrospinal fluid (CSF), and urine (Mastrogiorgio et al., 2021; Donti et al., 2016). Importantly, the S-Ado/SAICAr ratio in CSF correlates with disease severity, where lower ratios are observed in individuals with the neonatal phenotype, while higher ratios correspond with milder presentations. Emerging evidence suggests that SAICAr may trigger neuroinflammatory responses, although the exact mechanisms remain unclear (Jia et al., 2015; Jurecka et al., 2012; Dutto et al., 2022; Zulfiqar et al., 2013).

Evidence suggests that SAICAr accumulation leads to complement system activation, particularly via the alternative pathway, contributing to neuroinflammation, immune cell recruitment, and disruption of the blood-brain barrier. This pro-inflammatory cascade may play a key role in ADSLDD-associated neurodegeneration. This review synthesizes current knowledge of ADSLDD, emphasizing how metabolic dysfunction and immune dysregulation intersect, and evaluates the potential of complement-targeted therapies to mitigate disease progression.

Although no FDA-approved therapies currently exist for ADSLDD, several therapeutic strategies have been explored with varying degrees of success. These include dietary interventions such as D-ribose and uridine supplementation aimed at bypassing defects in purine synthesis, as well as supplementation with S-adenosylmethionine (SAMe) or other antioxidants, and the ketogenic diet (Jurecka et al., 2015). Experimental approaches such as gene therapy and enzyme replacement are in early preclinical stages. Moreover, metabolite-reducing strategies, including substrate scavenging or inhibition of SAICAr accumulation, are being investigated as targeted interventions (Mastrogiorgio et al., 2021; Dutto et al., 2022). Despite limited clinical evidence, some patients with milder phenotypes have shown partial improvement in seizures and behavior with combined metabolic support (Pérez-Dueñas et al., 2012; van Werkhoven et al., 2013; Jurecka et al., 2008a).This lack of curative treatments highlights the urgent need to understand how metabolic toxicity, immune activation, and genotype-phenotype correlations shape disease progression.

In the following sections, we begin by outlining the general mechanisms of complement activation, including the classical, lectin, and alternative pathways. We then examine how these pathways contribute to immune dysregulation in ADSLDD, particularly in the context of SAICAr accumulation. Special attention is given to the role of complement effector molecules such as C5a, and their relevance to blood-brain barrier disruption. We conclude with a discussion of existing knowledge gaps and future directions for targeted therapeutic interventions.

## 2 Complement activation

The complement system is a vital component of immune defense, consisting of approximately 40–50 proteins predominantly synthesized in the liver, with additional local production by various immune and non-immune cells such as monocytes, macrophages, dendritic cells, lymphocytes, adipocytes, fibroblasts, and epithelial cells (Magnusen and Pandey, 2024). This system functions through a tightly regulated cascade, providing critical roles in pathogen elimination, immune complex clearance, and modulation of inflammatory responses, all while maintaining homeostasis and minimizing host tissue damage (van Essen et al., 2023; Mulfaul et al., 2022). Complement activation proceeds through three distinct but converging pathways, (e.g., classical, lectin, and alternative pathways) (Magnusen and Pandey, 2024; Shehab El-Din et al., 2021; Lu et al., 2023; Pangburn, 2023). Each pathway is triggered by different recognition events but converges on the cleavage of C3 and ultimately C5, leading to the generation of potent effectors such as the anaphylatoxins (C3a, C4a, C5a), the opsonin C3b, and the terminal membrane attack complex (MAC or C5b-9) that mediates direct cytolysis (Magnusen and Pandey, 2024; Oechtering et al., 2025; Kemper et al., 2023; West and Kemper, 2023; Desai et al., 2023). Below, we describe the key features of each activation route and their molecular mechanisms of action.

## 3 Classical pathway of complement activation

**Fig. 1:**
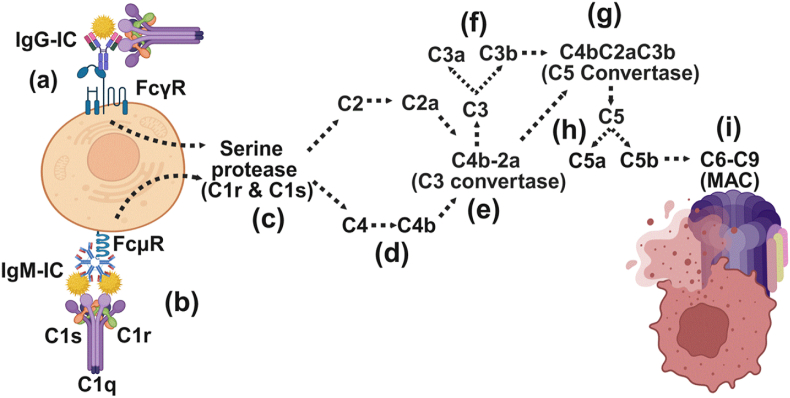
Classical pathway of complement activation.

The classical pathway is initiated when the C1 complex, comprising C1q, C1r, and C1s, binds to the Fc region of IgM or IgG antibodies that are either part of immune complexes or attached to antigens on damaged host cells. This binding activates the serine proteases C1r and C1s, which subsequently cleave complement components C4 and C2, forming the C3 convertase (C4b2a). This enzyme complex cleaves C3 into C3a, a potent anaphylatoxin, and C3b, which promotes opsonization and immune clearance. Addition of another C3b molecule to the convertase yields the C5 convertase (C4b2aC3b), which cleaves C5 into the chemoattractant C5a and C5b, the latter initiating assembly of the MAC, ultimately leading to target cell lysis (Fig. 1a–i).

## 4 Lectin pathway of complement activation

**Fig. 2:**
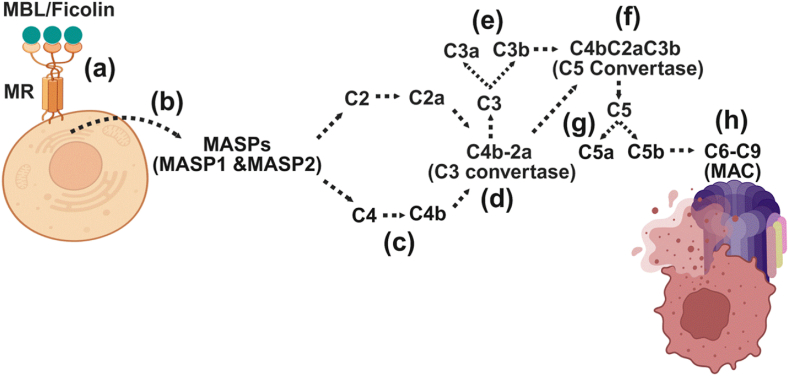
Lectin pathway of complement activation.

The lectin pathway shares many features with the classical pathway but is initiated by pattern recognition molecules such as mannose-binding lectin (MBL) or ficolins, which bind to carbohydrate motifs (e.g., mannose or N-acetylglucosamine) on microbial surfaces or stressed host cells. These recognition events activate MBL-associated serine proteases (MASP 1 and MASP2), which cleave C4 and C2 to generate the classical C3 convertase (C4b2a). As with the classical pathway, this leads to the production of C3a and C3b, followed by C5 convertase formation (C4b2aC3b), C5 cleavage, and MAC assembly (Fig. 2a-h)

## 5 Alternative pathway of complement activation

**Fig. 3:**
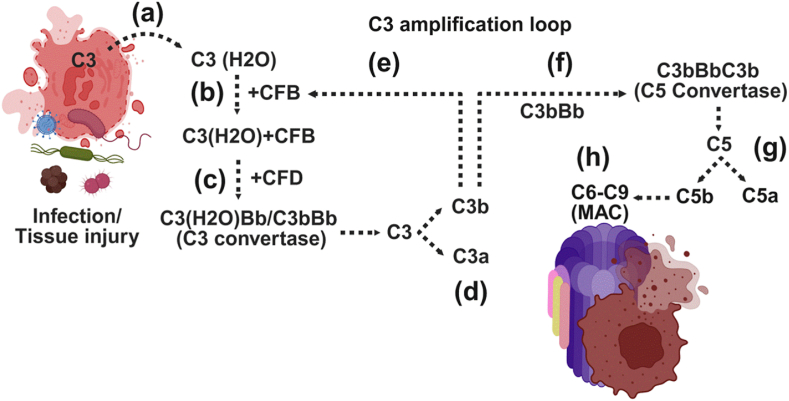
Alternative pathway of complement activation.

The alternative pathway is unique in that it can be spontaneously activated through the low-level hydrolysis of C3 to C3(H_2_O), which can bind factor B (CFB). Factor D (CFD) then cleaves CFB into Ba and Bb, forming the fluid-phase C3 convertase C3(H_2_O)Bb. Once surface-bound, C3b can initiate a positive feedback loop by forming the surface-bound C3 convertase (C3bBb), which amplifies C3 cleavage. The addition of a second C3b molecule generates the C5 convertase (C3bBbC3b), resulting in C5 cleavage into C5a and C5b, and triggering MAC assembly. This pathway plays a critical role in amplifying complement activation on pathogen or altered host cell surfaces (Fig. 3a–h).

## 6 Convergence and pathological implications in ADSLDD

**Fig. 4:**
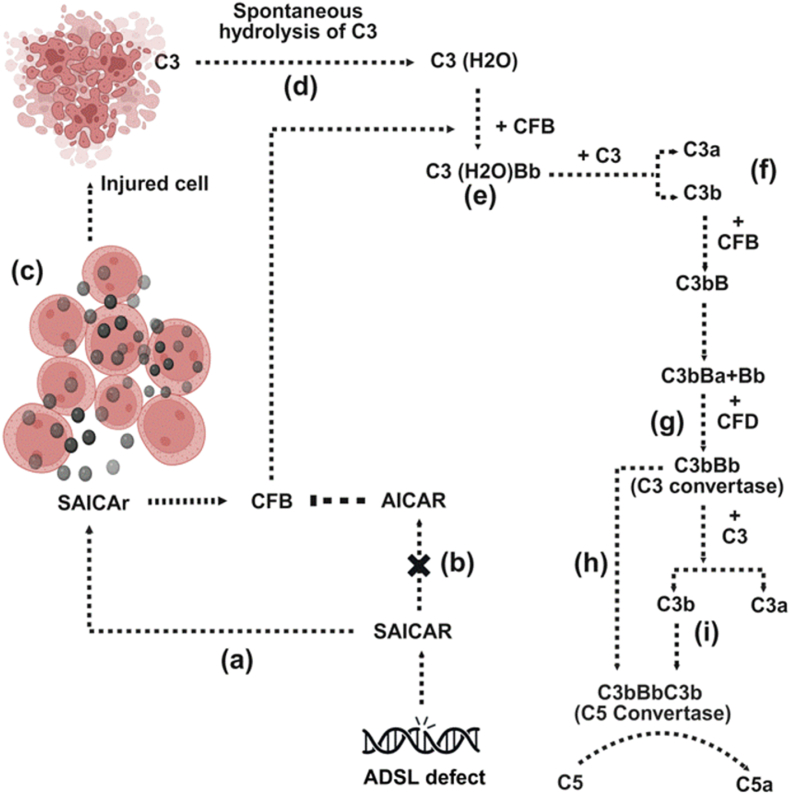
Activation of the alternative complement pathway in Adenylosuccinate lyase deficiency disorder.

In the context of ADSLDD, excess of SAICAR-induces SAICAr that disrupts the normal production of AICAR. AICAR is known to inhibit the expression of complement factor B (CFB)(Chung et al., 2017). CFB plays a role in activating the alternative pathway of complement activation (Magnusen and Pandey, 2024; Laskowski et al., 2018; Kerr et al., 2013). When AICAR production is compromised due to genetic deficiency of the ADSL, there is an accumulation of SAICAR, which leads to increased levels of its dephosphorylated form, SAICAr. This shift results in the loss of AICAR's ability to inhibit complement factor B. This disruption creates a harmful feedback loop where elevated SAICAr levels interact with leukocytes, provoking cellular injury. This injury catalyzes the spontaneous hydrolysis of complement component C3 into C3(H2O), which then binds to complement factor B, forming the enzyme complex C3(H2O)Bb. This complex cleaves additional C3 into C3a and C3b, propelling the activation of the complement cascade. The generated C3b binds to complement factor B, enabling complement factor D to cleave B into its active forms, Ba and Bb, thereby producing the potent C3 convertase (C3bBb). The amplification of this pathway is profound; the C3bBb complex further engages more C3b to form the C5 convertase (C3bBbC3b), resulting in the cleavage of C5 into C5a. This series of events illustrates not only the biochemical ramifications of ADSLDD but also illustrates a critical vulnerability in the immune response (Fig. 4a–i).

## 7 Complement effector function in ADSLDD

Complement 3a (C3a) is a 9 kDa peptide, comprised of 77 amino acids arranged in a distinct structure stabilized by three disulfide bridges. This configuration is essential for its biological activity and interaction with its C3a receptor (C3aR) (Klos et al., 2013). C3aR is expressed in numerous organs and immune cell types, highlighting its importance in immune responses (Magnusen and Pandey, 2024). The binding of C3a to C3aR mediates multiple physiological and pathological effects and is implicated in conditions such as fibrosis, heart failure, liver damage, lung injury, and nephropathy (Magnusen and Pandey, 2024).

Complement 5a (C5a) serves as an anaphylatoxin and chemoattractant, playing a pivotal role in inflammation and host defense (Jongerius et al., 2007; Zhang et al., 2010; Köhl et al., 2006; Bestebroer et al., 2010; Fritsche et al., 2010). Structurally, C5a is a 15 kDa glycoprotein derived from the larger C5 protein. It interacts with two receptors: C5aR1, which mediates pro-inflammatory signaling, and C5aR2, which acts as a decoy receptor to modulate the inflammatory response (Magnusen and Pandey, 2024; Zhang et al., 2010; Köhl et al., 2006; Weaver et al., 2010). C5aR1 is expressed on both immune and non-immune cells, facilitating the recruitment and activation of immune responses in tissues including liver, spleen lungs, and brain (Magnusen and Pandey, 2024).

The complement proteins C3a and C5a play crucial roles in activating immune cells, such as monocytes, macrophages, dendritic cells, and T cells, as well as neuroglial cells like astrocytes and microglia (Magnusen and Pandey, 2024; Pandey, 2013, 2023; Pandey et al., 2017). They trigger signaling cascades, i.e., Ak strain transforming/protein kinase B (Akt/PKB), mitogen-activated protein kinase (MAPK), extracellular signal-regulated kinases (ERK), and nuclear factor kappa-light-chain-enhancer of activated b cells (NF-κB) that promote the release of pro-inflammatory cytokines, including IFNγ, IL1β, TNFα, IL6, and IL-17 (Magnusen and Pandey, 2024)^,^ (Pandey et al., 2017)^,^ (Luo et al., 2022; Gong et al., 2022; Liu et al., 2018; Fattahi et al., 2017). These cytokines, along with their downstream signaling pathways, play a central role in sustaining chronic inflammation and driving progressive tissue injury. Their dysregulation has been implicated in a wide range of pathological conditions, including autoimmune diseases, ischemia-reperfusion injuries, and both renal and cardiovascular disorders (Neumann et al., 2002; Grant et al., 2002; Belmont et al., 1986; Peng et al., 2012; Hu et al., 2021; Monsinjon et al., 2001; Genest et al., 2022; Łukawska et al., 2018; Zhang et al., 2017; Bantis et al., 2021; Oksjoki et al., 2007; Speidl et al., 2010; Friščić et al., 2021).

Similarly, in lysosomal storage diseases such as Gaucher, Fabry, and Niemann-Pick type C, genetic deficiencies in specific enzymes lead to the harmful accumulation of glycolipid substrates. This accumulation activates the complement system, resulting in the overproduction of complement proteins C3a and C5a. The binding of C3a to its receptor (C3aR) and C5a to C5a receptor 1 (C5aR1) initiates a cascade of downstream signaling, such as AKT and the resulting inflammatory responses characterized by the activation of macrophages, dendritic cells, and T cells, increased production of pro-inflammatory cytokines, including IFNγ, IL1β, TNFα, IL6, IL12, and IL17 that contribute to visceral and central nervous system damage (Magnusen and Pandey, 2024; Pandey, 2023; Pandey et al., 2017, 2018; Klein et al., 2020; Trivedi et al., 2022; Serfecz et al., 2021).

Beyond their classical roles in peripheral immunity, complement proteins are increasingly recognized as key regulators of central nervous system (CNS) function, particularly in modulating synaptic pruning, neuroinflammation, and maintaining blood brain barrier (BBB) integrity, which are vital for brain development and cognitive health (Nimmo et al., 2024; Lee et al., 2019; Gorelik et al., 2017). More recently, growing interest has focused on complement systems in neurodevelopmental and neuropsychiatric disorders. Dysregulation of complement activation has been implicated in the pathophysiology of several mental health conditions, including anxiety, depression, schizophrenia, and autism spectrum disorders. Notably, the classical pathway initiator C1q has been implicated in excessive synaptic pruning during adolescence, a critical window for brain maturation that also marks increased susceptibility to psychiatric disorders such as schizophrenia and anxiety (Westacott and Wilkinson, 2022; Druart and Le Magueresse, 2019; Yousefpour et al., 2025). Furthermore, elevated expression of central and peripheral complement components C3 and C4 has been reported in patients with generalized anxiety disorder, major depressive disorder, and schizophrenia (Shatri et al., 2023; Pillai, 2022; Sekar et al., 2016a).

Abnormalities in C1q and C4 are frequently associated with increased production of the anaphylatoxins C3a and C5a, which function as potent chemoattractant and immunomodulatory mediators across both peripheral and central tissues (Chung et al., 2023; Absinta et al., 2021; Reichhardt and Meri, 2018; Magdalon et al., 2020; Stennett et al., 2023; Oechtering et al., 2024; Negro-Demontel et al., 2024). Upon binding to their respective receptors, C3aR and C5aR1, these complement-derived molecules play a central role in orchestrating immune responses. In addition to their classical function in directing leukocyte recruitment to sites of infection or tissue damage, C3a and C5a also drive the production of a broad array of pro-inflammatory cytokines thereby amplifying both systemic and neuroinflammatory signaling cascades (Yadav et al., 2023; Schanzenbacher et al., 2023; Duan et al., 2023; Karsten et al., 2012). These pro-inflammatory cytokines, (e.g., IFNγ, IL1β, IL6, IL17, and TNFα) are frequently elevated in a wide range of inflammatory and neurodegenerative conditions (Magnusen and Pandey, 2024; Pandey, 2022, 2023; Sadowska et al., 2015; Geng et al., 2018; Chen et al., 2022; Setiadi et al., 2019). Their sustained presence has been strongly implicated in the disruption of the BBB, a critical event that facilitates the infiltration of peripheral immune cells into the CNS. This breach of immune privilege not only amplifies neuroinflammatory signaling but also accelerates neuronal injury and disease progression. Persistent C3a/C3aR and C5a/C5aR1 signaling thus contributes to a vicious cycle of BBB breakdown and chronic inflammation, key features underpinning the pathogenesis of numerous CNS disorders, including Alzheimer's disease (AD), systemic lupus erythematosus (SLE), stroke, amyotrophic lateral sclerosis (ALS), and neuromyelitis optica spectrum disorder (NMOSD)(Mahajan et al., 2015; Jacob et al., 2010a, 2011; Landlinger et al., 2015; Woodruff et al., 2006; Farkas et al., 2003; Yuan et al., 2017; Kim et al., 2008; Lee et al., 2017; Piatek et al., 2018; Dhillon, 2018; Carvalho et al., 2022; Manoj Kumar, 2025).

Excessive activation of the C5a–C5aR1 axis, particularly under chronic stress conditions, has been linked to microglial cell activation, hippocampal neurodegeneration, and the development of anxiety-like behaviors in animal models (Chen et al., 2024). These findings are particularly relevant in the context of ADSLDD, where elevated levels of the purine intermediate SAICAr are associated with a constellation of neurological and psychiatric symptoms. These include developmental delay, epilepsy, autism-like behaviors, and mood disturbances such as irritability, agitation, and anxiety (Mouchegh et al., 2007; Dutto et al., 2022; Van den Bergh et al., 1993; Kmoch et al., 2000; Jurecka et al., 2008b; Zikanova et al., 2010; Marie et al., 2000; Georges and Berghe, 1984; Maaswinkel-Mooij et al., 1997; Lee and Colman, 2007; Palenchar et al., 2003).

SAICAr has been shown to activate key intracellular signaling pathways, including AKT, MAPK, and ERK, leading to the induction of pro-inflammatory cytokines (Keller et al., 2012, 2014; Yan et al., 2016). Interestingly, these same signaling pathways are also activated downstream of C3a–C3aR and C5a–C5aR1 engagement (Magnusen and Pandey, 2024; Pandey, 2023; Pandey et al., 2017) suggesting a convergence of SAICAr accumulation and complement signaling on common molecular circuits involved in neuroinflammation and neuronal injury. Within the hippocampus, this convergence may exacerbate neuronal dysfunction by impairing neurogenesis and promoting oxidative and inflammatory stress (Dutto et al., 2022; Stone et al., 1998).

Of particular interest is the role of AICAR (5-aminoimidazole-4-carboxamide ribonucleotide), a downstream metabolite normally regulated by ADSL activity. AICAR has been reported to inhibit complement factor B expression in retinal pigment epithelial cells, thereby suppressing alternative pathway activation and reducing the production of downstream inflammatory mediators such as C3a and C5a (Chung et al., 2017; Brown et al., 2023). In preclinical models, AICAR and its analogs have also shown anti-inflammatory effects, including the suppression of NF-κB signaling, inhibition of T-cell proliferation, and reduction of pro inflammatory cytokines such as IFNγ, TNFα, IL1β, IL6, and IL17 in models of endotoxin-induced uveitis and experimental autoimmune uveoretinitis (Hall et al., 2018; Suzuki et al., 2011, 2012). Furthermore, AICAR has demonstrated protective effects against inflammation-induced muscle wasting, underscoring its broader immunomodulatory potential (Hall et al., 2018).

Our synthesis of the current literature reveals a compelling mechanistic insight: AICAR, which may be depleted in the setting of ADSL deficiency, possesses intrinsic inhibitory activity against complement factor B and is a key regulator of anti-inflammatory signaling. In ADSLDD, the pathological accumulation of SAICAr may be paralleled by a functional deficiency of AICAR, tipping the balance toward unchecked complement activation. This imbalance may perpetuate a self-amplifying cycle of neuroinflammation, driven by complement overactivation and pro-inflammatory cytokine production.

**Fig. 5:**
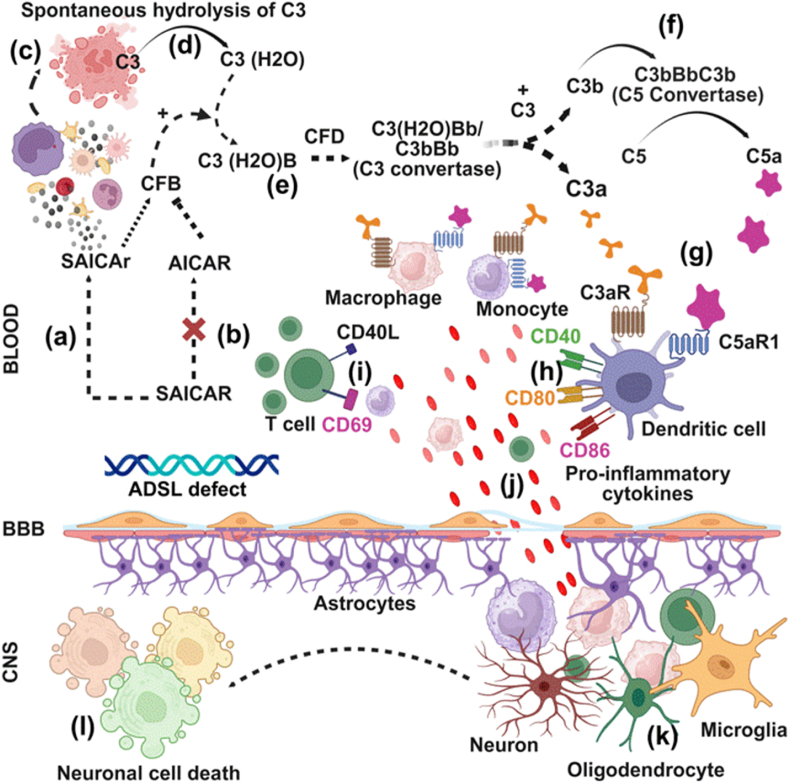
Effector functions of complement activation products drive the development of neuroinflammation in Adenylosuccinate lyase deficiency disorder.

Building on these findings, we offer a different understanding of the mechanisms underlying ADSLDD, particularly emphasizing the disruptive role of SAICAr in essential metabolic pathways. The deficiency of the ADSL enzyme leads to a cellular accumulation of SAICAR and its dephosphorylated form, SAICAr in the circulatory system, which impairs the synthesis of AICAR (Fig. 5a–c). Notably, this metabolic imbalance alters the dynamics of SAICAr, creating a threshold that activates complement factor B (Fig. 5b), which initiates aberrant complement activation via the alternative pathway, resulting in increased production of terminal complement components, such as C3a and C5a (Fig. 5d–f).

When C3a and C5a bind to their respective receptors, they trigger a cascade of inflammatory responses (Fig. 5g). This leads to the upregulation of costimulatory molecules (e.g., CD40, CD80, and CD86) on phagocytes including monocytes, macrophages, and dendritic cells, as well as stimulatory molecules (e.g., CD40L and CD69) on T cells resulting in excessive production of pro-inflammatory cytokines (Fig. 5h-j).

The elevated levels of these inflammatory mediators compromise the integrity of the blood-brain barrier, allowing for the infiltration of circulating leukocytes, such as monocytes, macrophages, and T cells into the central nervous system. This infiltration fosters detrimental interactions with various neuronal cells, including astrocytes, neurons, oligodendrocytes, and microglia (Fig. 5k). Ultimately, these interactions contribute to cellular damage and neurotoxicity, which are hallmarks of ADSLDD (Fig. 5l).

Although AICAR remains a valuable experimental tool for dissecting anti-inflammatory signaling and metabolic regulation, its clinical translation is limited by poor stability, low bioavailability, and dose-dependent adverse effects (Hall et al., 2018; Paré and Jasmin, 2017; Marangos et al., 1990). In parallel, converging evidence from animal models and human studies demonstrates that dysregulated complement activation is a shared and pathogenic feature across a wide spectrum of neurological and neuropsychiatric disorders. In degenerative conditions such as AD, ALS, Huntington's disease (HD), and Parkinson's disease (PD), overactivation of components like C1q, C3, and C5a drives aberrant synaptic pruning, fuels microglia-mediated neuroinflammation, and in some cases induces dopaminergic neuron loss. In neurodevelopmental and psychiatric disorders including autism spectrum disorder (ASD), bipolar disorder (BD), schizophrenia, and attention-deficit/hyperactivity disorder (ADHD) altered complement profiles are observed both centrally and peripherally, with disease-specific patterns of increased or decreased C1q, C3, C4, C5, MASP-1, and C5b-9 levels correlating with behavioral phenotypes, social deficits, or cognitive impairment. In autoimmune-mediated neurological diseases such as generalized myasthenia gravis (GMG), Guillain-Barré syndrome (GBS), NMOSD, and SLE, complement activation products directly damage neuromuscular junctions, astrocytes, or myelin, often in proportion to disease activity. Notably, targeted inhibition of key complement mediators (C3, C5, C5aR1) or upstream activators (C1q, C2) have shown consistent neuroprotective effects in preclinical models and early-phase clinical trials across multiple conditions, including AD, ALS, GBS, NMOSD, stroke, and even certain psychiatric models (Table 1).

**Table 1:** Contribution of the complement system to neuropathology and behavioral impairment in neurological disease.

Disease	Animal models	Human subjects	Reference
Alzheimer's disease	Tg2576 and Arctic48 transgenic mice (on C57BL/6J or 129S6 background) treated with C5aR1 inhibitor (PMX-205) or C5a vaccine show reduced neuroinflammation and improved memory.	Elevated C5a in serum/CSF correlates with cognitive decline and proinflammatory cytokines in AD.	(Landlinger et al., 2015; Farkas et al., 2003; Schartz et al., 2024; Fonseca et al., 2009; Yang et al., 2024)
Amyotrophic lateral sclerosis	hSOD1G93A transgenic mice (C57BL/6J background, ∼25 SOD1 copies) treated with PMX-205 show reduced neuroinflammation, delayed motor deficits, and improved survival.	Elevated plasma C5a and C5b-9 in ALS patients; C5 mAbs (ravulizumab) reduce relapse risk and clinical worsening.	(Lee et al., 2017; Genge et al., 2023; Mantovani et al., 2014)
Anxiety and depression	C3aR signaling in hippocampus drives anxiety-like behavior in C57BL/6 mice with inflammatory pain model.	In SLE patients, low serum C3/C4 correlates with increased anxiety severity.	(Shatri et al., 2023; Zhang et al., 2025)
Attention-deficit/hyperactivity disorder	NR	In patients with ADHD, plasma levels of C4b were decreased, while serum levels of C5b-9 were elevated. Presence of elevated C5b-9 was associated with an increased risk of ADHD.	(Al-Hasnawi et al., 2024; Warren et al., 1995)
Autism spectrum disorder	In ASD mouse models, C3 knockdown in the prefrontal cortex (C57BL/6) induced social deficits and increased repetitive behaviors without overt neuronal loss. Mice deficient in C1q or C3 exhibited impaired synaptic pruning and ASD-like behaviors, whereas C4 overexpression led to social impairments.	In ASD patients, postmortem cortical tissue shows reduced C1q, C3, and C4, but elevated C2, C5, and MASP-1 levels, with the latter correlating with ADI-R scores. Serum studies reveal mixed patterns, with some components (e.g., C1q, C3) decreased and others (e.g., C2, C5, CFHR1) elevated, suggesting complex, region-specific complement dysregulation.	(Fagan et al., 2017; Gunaydin et al., 2025; Mou et al., 2022; Warren et al., 1994; Corbett et al., 2007; Shen et al., 2018; Momeni et al., 2012; Nardone et al., 2014)
Bipolar disorder	NR	In patients with BD, serum levels of multiple complement components including C1q, C4, CFB, CFH, properdin, C3a, C5a, and C5b-9 were elevated. Higher levels of these components correlated with reduced gray matter volume in the medial orbitofrontal cortex and middle cingulum, as well as decreased cortical thickness in the left precentral and left superior frontal gyri. In contrast, postmortem brain tissue from BD patients showed reduced expression of C1q, C3, C1r, C1s, and CFB, suggesting a peripheral–central divergence in complement activation and regulation.	(Reginia et al., 2018; Sager et al., 2025)
Generalized myasthenia gravis	Rats treated with cobra venom factor (to deplete complement) during the acute phase of active or passive EAMG were protected from NMJ pathology. Mice lacking complement regulators (DAF and CD59) showed worsened NMJ damage, while C3^−/−^ or C4^−/−^ mice exhibited complete resistance to weakness. Treatment with C5mAbs in both mouse and rat EAMG models preserved NMJ integrity and improved motor outcomes.	In patients with MG, NMJs exhibit deposition of C3 and MAC, consistent with complement-mediated injury. Terminal complement components are elevated in patient sera, which can induce lysis of cultured myotubes in vitro. Clinical studies have shown that C5 monoclonal antibody therapy (eculizumab) improves muscle strength in patients with refractory, anti-AChR antibody–positive GMG.	(Dhillon, 2018; Kusner and Kaminski, 2012; Lennon et al., 1978; Tüzün et al., 2003; Martinez Salazar et al., 2025; Murai et al., 2019; Muppidi et al., 2019; Mantegazza et al., 2020; Siddiqi et al., 2021; Nakano and Engel, 1993; Sahashi et al., 1980; Engel et al., 1979, Engel et al., 1977; Barohn and Brey, 1993; Ashizawa and Appel, 1985; Chamberlain-Banoub et al., 2006)
Guillain-Barré syndrome	In Balb/c mouse models of MFS and AMAN, both forms of GBS induced by anti-GQ1b antibodies therapeutic administration of C5mAbs (eculizumab) or anti-C1q mAbs markedly reduced disease severity and mitigated peripheral nerve injury. Similarly, in a GM1-sensitized rabbit model of AMAN, treatment with the complement inhibitor nafamostat mesylate diminished C3c and C5b-9 deposition at the nodes of Ranvier and preserved voltage-gated sodium channel expression, thereby maintaining nodal structural integrity and functional conduction capacity.	In patients with GBS, autoantibodies against gangliosides (e.g., GM1, GD1a, GT1b) were shown to activate complement, resulting in deposition of C3 and C5b-9 on nerves and complement-mediated neuronal cell lysis in vitro. Clinical trials of complement inhibitors eculizumab (C5 blockade, Phase 2) and ANX005 (C1q blockade, Phase 1b) demonstrated safety and preliminary signs of efficacy.	(Campbell et al., 2022; McGonigal et al., 2016; Phongsisay et al., 2008; Halstead et al., 2008; Hafer-Macko et al., 1996a, Hafer-Macko et al., 1996b; Davidson et al., 2017; Misawa et al., 2018)
Huntington's disease	In the zQ175 knock-in mouse model of HD (heterozygous CAG expansion), elevated levels of C1q and C3 were detected in the striatum and motor cortex, with C1q predominantly expressed by microglia and C3 by ependymal cells lining the lateral ventricles; increased association of C3 with corticostriatal synapses highlights early complement-mediated synaptic alterations that parallel initial cognitive deficits and synapse loss. Systemic administration of a C1q-blocking antibody (ANX-M1) or genetic CR3 deficiency significantly reduced synaptic complement deposition, prevented synapse loss, and preserved cognitive function and synaptic integrity in this HD model.	Postmortem HD brains show elevated C1q, C3, iC3b, C4, C9 in striatum; CSF C3/iC3b levels correlate with disease burden; measured by CAP score. Humanized C1q mAbs (wANX005) phase 2a trial shows HD biomarker reduction.	(Wilton et al., 2023; Singhrao et al., 1999; Caron et al., 2022; Kumar et al., 2023)
Neuromyelitis optica spectrum disorder	NMOSD mouse model (C57BL/6J) spinal cord and optic nerve cultures exposed to NMO-IgG and complement develop hallmark pathology, including loss of AQP4 and GFAP and altered glutamate receptor expression. Deletion or pharmacological inhibition of C5aR1 with uses of C5aR antagonist (C5aRA) preserves AQP4 and GFAP expression, enhances motor function, increases NMDAR activity, and modulates glutamate neurotoxicity, highlighting the critical role of C5a/C5aR signaling in NMOSD pathogenesis.	Elevated serum/CSF C1-INH, C1s, C3/C4 ratio, CFH, and C5a; AQP4+IgG induces MAC-mediated astrocyte injury; C5 mAbs (eculizumab/ravulizumab) reduce relapses.	(Piatek et al., 2018; Li et al., 2022; Cakan et al., 2025; Hakobyan et al., 2017; Cho et al., 2024; Tong et al., 2022; Pittock et al., 2013, Pittock et al., 2019, [Bibr bib177][Bibr bib123])
Parkinson's disease	Wild-type (WT) C57BL/6 mice exposed to P + M show increased CR3/NLRP3 signaling and LC/NE neuron loss; CR3 ^−/−^ mice (C57BL/6) show protection against dopaminergic neuron loss and the development of motor deficits; CR3 inhibition blocks Nox2 activation.	CSF C3/Aβ42 ratios correlate with PD severity; brain iC3b and C9 elevated; α-synuclein induces complement-dependent toxicity in vitro.	(Hou et al., 2018, Hou et al., 2020; Wang et al., 2011; Loeffler et al., 2006; Gregersen et al., 2021)
Schizophrenia	In mouse models of schizophrenia, overexpression of C4A/B induces excessive synaptic pruning via the classical complement pathway, modeling inflammation-related mechanisms in schizophrenia. C1q^−/−^ (C57BL/6J) and C3^−/−^ (DBA/2J) mice show impaired synapse elimination, suggesting complement function in synaptic remodeling and schizophrenia pathophysiology.	In schizophrenia, particularly in chronic cases, elevated brain expression of C1QA,C1QB, C1R and CFB were observed; Patients with schizophrenia show increased levels of C1q containing immune complexes, often formed with IgG antibodies against dietary antigens like casein and gluten (indication of the classical pathway activation); structural variation in C4 genes contributes to schizophrenia risk through increased C4A and C4B expression, with risk alleles showing a strong correlation between elevated C4A and C4B levels in brain tissue and disease susceptibility. C4 protein localizes to synapses, dendrites, axons, and cell bodies; patients with schizophrenia exhibit elevated MBL-bound MASP-2 (indication of the lectin pathway activation) activity.	(Mou et al., 2022; Sager et al., 2025; Stevens et al., 2007; Nimgaonkar et al., 2017; Sekar et al., 2016b; Mayilyan et al., 2006; Severance et al., 2012)
Systemic lupus erythematosus	MRL/lpr lupus-prone mice treated with C5aR antagonist (C5aRA) show suppression of JNK, STAT1, ERK, Akt, and NF-κB pathways and preservation of BBB integrity.	SLE patients show brain inflammation with elevated C5a; complement dysregulation associated with neuropsychiatric symptoms.	(Mahajan et al., 2015; Jacob et al., 2010a, Jacob et al., 2010b; Buyon et al., 1992)
Stroke/intracerebral hemorrhage (ICH)	C3^−/−^ mice (C57BL/6J) show reduced infarct volume; reconstitution of C3 or activation of C3aR reverses protection; C5aR1 activation worsens outcomes via ERK1/2 and p38 MAPK; anti-C5, C5aR antagonists, and B4Crry improve outcomes in mouse and rat stroke models.	Plasma C5a elevated post-ischemia; carotid atherosclerosis patients show high alternative pathway markers (CFD or D, properdin, C3bBbP).	(Yuan et al., 2017; Kim et al., 2008; Alawieh et al., 2018; Mocco et al., 2006a, Mocco et al., 2006b; Costa et al., 2006; Louwe et al., 2025)

AChR (acetylcholine receptor), AD (Alzheimer's disease), ADHD (Attention-deficit/hyperactivity disorder), Akt/PKB (Ak strain transforming/protein kinase B), ALS (amyotrophic lateral sclerosis), AMAN (acute motor axonal neuropathy), ASD (Autism spectrum disorder), AQP4 (Aquaporin-4), BBB (blood–brain barrier), BD (Bipolar disorder), C (complement component), C3a (complement 3a), C3aR (C3a receptor), C5a (complement 5a), C5aR1 (C5a receptor 1), CAP score (CAG-age product score, a disease burden metric in HD), CF (complement factor), CSF (cerebrospinal fluid), EAMG (experimental autoimmune myasthenia gravis), ERK1/2 (extracellular signal-regulated kinases 1 and 2), GBM (Guillain-Barré syndrome), GFAP (glial fibrillary acidic protein), GMG (generalized myasthenia gravis), HD (Huntington's disease), ICH (intracerebral hemorrhage), iC3b (inactivated C3b), JNK (Jun N-terminal kinase), LC/NE (locus coeruleus noradrenergic), MAPK (mitogen-activated protein kinase), MASP (MBL-associated serine protease), MBL (mannose binding lectin), M (maneb; fungicide used to control plant diseases), MFS (Miller Fisher syndrome), NF-κB (nuclear factor kappa-light-chain-enhancer of activated b cells), NLRP3 (NLR; NOD-like Receptor family, pyrin domain containing 3), NMJs (neuromuscular junctions), NMOSD (neuromyelitis optica spectrum disorder), NR (Not reported), P (paraquat; highly toxic chemical used as a herbicide), p38 MAPK (p38 mitogen-activated protein kinases), PD (Parkinson's disease), SLE (systemic lupus erythematosus), and STAT1 (signal transducer and activator of transcription 1).

Taken together, these findings position the complement cascade not as a disease-specific anomaly but as a central pathological amplifier across neurological injury and psychiatric dysfunction. For ADSLDD and related neuroinflammatory disorders, a dual therapeutic approach restoring AICAR activity to bolster endogenous anti-inflammatory pathways while simultaneously modulating complement activation could attenuate complement-driven inflammation, stabilize the BBB, and preserve neurobehavioral function.

## 8 Discussion

This review highlights a novel mechanistic link between metabolic dysfunction and immune activation in ADSLDD. We summarize current evidence showing that accumulation of SAICAr, a toxic purine intermediate, is not merely a biomarker of disease severity but a direct activator of the alternative complement pathway, contributing to chronic neuroinflammation. We further describe how reduced levels of AICAR, a downstream metabolite normally produced by ADSL defects, may impair regulatory control over complement activity. Together, these findings point to a metabolically driven inflammatory cascade that contributes to the neurological deterioration observed in ADSLDD. By synthesizing metabolic and immunological insights, this review identifies complement modulation as a promising therapeutic target and suggests future directions for integrative treatment strategies.

Although patients with ADSLDD typically maintain normal serum levels of purine nucleotides, they show marked accumulation of dephosphorylated substrates of ADSL, most notably S-Ado and SAICAr. Among these, SAICAr has been increasingly recognized for its potential neurotoxicity and its association with a broad spectrum of neurobehavioral disturbances. Clinical manifestations can range from seizures, developmental delay, and intellectual disability to behavioral symptoms such as aggression, irritability, social withdrawal, and mood dysregulation, reflect the profound cognitive and emotional impairment associated with this disorder (Cutillo et al., 2024; Wang et al., 2022; Georges and Berghe, 1984).

Experimental depletion of ADSL in human cellular models results in phenotypic abnormalities, including heightened DNA damage signaling and delayed cell cycle progression effects that have been linked specifically to SAICAr accumulation or loss of ADSL enzymatic activity (Dutto et al., 2022). Both DNA damage signaling and delayed cell cycle progression interact with innate immune pathways to drive sustained inflammation (Muralidharan and Mandrekar, 2013; He et al., 2024) However, despite these advances, the molecular mechanisms by which ADSL dysfunction leads to sustained neuroinflammation and neurodegeneration remain insufficiently defined.

Our findings point to a novel and compelling link between SAICAr accumulation and activation of the alternative pathway of the complement system a pathway known for its low-level basal activity and rapid amplification in response to central nervous system injury or cellular stress (Brennan et al., 2012; Yarmoska et al., 2021). We propose that excess SAICAr may interact with immune cells, particularly leukocytes, triggering the hydrolysis of complement component C3 and initiating complement activation. This results in the generation of pro-inflammatory fragments such as C3a and C5a, which are potent mediators of inflammation (Magnusen and Pandey, 2024). Their engagement with respective receptors on glial and immune cells may drive sustained neuroinflammatory cascades, contributing to the progressive neuronal damage and clinical deterioration observed in ADSLDD patients. This connection offers an opportunity to deepen our understanding of the disease mechanism and identify new therapeutic strategies. By clarifying these connections, we could open the door to more precise and effective approaches for managing ADSLDD and its complications.

Activation of the C3a–C3aR and C5a–C5aR1 signaling axes initiates a complex cascade of immune responses that extends beyond microglial activation to include the release of pro-inflammatory cytokines, establishing a self-amplifying cycle of neuroinflammation within the brain (Magnusen and Pandey, 2024; Pandey, 2023; Sadowska et al., 2015; Geng et al., 2018; Chen et al., 2022; Setiadi et al., 2019; Magnusen et al., 2021; Hatton and Pandey, 2022; Marsili et al., 2023). This sustained inflammatory milieu not only disrupts neural homeostasis but also contributes to progressive tissue damage (Mahajan et al., 2015; Jacob et al., 2010a; Landlinger et al., 2015; Woodruff et al., 2006; Farkas et al., 2003; Yuan et al., 2017; Kim et al., 2008; Lee et al., 2017; Piatek et al., 2018; Dhillon, 2018; Carvalho et al., 2022; Chen et al., 2024; Propson et al., 2021; Cook, 2019; Qi et al., 2025; Pekna et al., 2021; Litvinchuk et al., 2018; Tang et al., 2024; Tan et al., 2024). Importantly, these same complement pathways have been implicated in a range of behavioral impairments such as neuropathic pain, memory deficits, motor abnormalities, anxiety or depression-like phenotypes, irritability, and agitation (Mahajan et al., 2015; Jacob et al., 2010a; Landlinger et al., 2015; Woodruff et al., 2006; Farkas et al., 2003; Yuan et al., 2017; Kim et al., 2008; Lee et al., 2017; Piatek et al., 2018; Dhillon, 2018; Carvalho et al., 2022; Chen et al., 2024; Propson et al., 2021; Cook, 2019; Qi et al., 2025; Pekna et al., 2021; Litvinchuk et al., 2018; Tang et al., 2024; Tan et al., 2024). Such manifestations are particularly relevant in disorders like ADSLDD, where metabolic dysfunction converges with immune dysregulation to exacerbate cognitive and psychiatric symptoms (Moro et al., 2023; Mastrogiorgio et al., 2021; Mastrangelo et al., 2019; Ciardo et al., 2001).

To date, there is no disease-modifying treatment for ADSLDD. Some strategies, such as D-ribose and uridine supplementation, have been proposed to enhance purine synthesis, but results have been inconsistent (Salerno et al., 1999). More recently, S-adenosylmethionine has shown minimal evidence of therapeutic benefit (van Werkhoven et al., 2013). Seizure control remains a mainstay of symptomatic treatment, with polypharmacy often required due to refractory epilepsy. A range of anti-seizure medications (e.g., phenobarbital, valproic acid, levetiracetam, carbamazepine) and the ketogenic diet have been trialed with variable success (Jurecka et al., 2015; Yudkoff et al., 2007). However, none of these treatments address the underlying immunometabolic dysregulation.

Given the growing availability of agents targeting key complement components including C1q, C3, C5, C5a, and C5aR1 (Table 2), there is strong rationale to explore their use in ADSLDD, where metabolic insufficiency and immune overactivation converge to drive pathology. Several of these agents are already FDA-approved or in advanced clinical development, offering a rapid path to translation. By intervening at critical nodes in the cascade, complement modulation could dampen neuroinflammation, stabilize the BBB, and protect neural circuits, leading to perceptible cognitive and behavioral benefits. Building on this premise, the next immediate steps include validating complement activation and immune perturbations in patient-derived samples and disease-relevant models measuring C3a,C3b C5a, C5b, and downstream cytokines in plasma and CSF, alongside transcriptomic and proteomic profiling of iPSC-derived microglia and neurons. In parallel, testing the efficacy of available C3 inhibitors, C5 mAbs, and C5aR1 targeting agents in preclinical systems will help define therapeutic potential. Together, these studies could provide the mechanistic and proof-of-concept foundation for early-phase trials, bringing us closer to targeted interventions for ADSLDD and potentially other metabolically driven neurodevelopmental disorders where complement acts as a pathological amplifier.

**Table 2:** Clinically available therapeutic agents for complement-mediated human diseases.

Condition	Pathophysiology	Therapeutic Options	Reference
Cold agglutinin disease	In CAD, IgM antibodies that target RBC antigens activate C1q, starting both the classical and alternative pathways. This leads to RBC destruction (intravascular hemolysis) through MAC. It also causes C3b, iC3b, or C3d to stick to RBCs, leading to their destruction in the liver (extravascular hemolysis).	C1s mAb (sutimlimab/enjaymo)	(Broome, 2023; Berentsen et al., 2022; Reid and Fedutes Henderson, 2024)
Age-related macular degeneration	AMD is linked to rare gene changes in complement proteins (like C3, C9, CFB, CFI, CFH, CD46), higher activity in the alternative pathway, damage to retinal cells and photoreceptors through MAC, and increased immune cell activity in the eye (via C3aR and C5aRs).	C3 inhibitor (pegcetacoplan/enjaymo)	(Pfau et al., 2022; Liao et al., 2020)
Paroxysmal nocturnal hemoglobinuria	In PNH, the absence of regulators like DAF/CD55 and MAC-IP/CD59 leads to increased activity in the Alternative Pathway. This causes MAC-mediated destruction of RBCs at night (intra vascular hemolysis) and C3b, iC3b, or C3d to deposit on RBCs, leading to their destruction in the liver (extra vascular hemolysis).	C3 inhibitor (pegcetacoplan/enjaymo), C5mAb (eculizumab) and ravulizumab/ultomiris)	(Chan et al., 2025; Hoy, 2021; McKeage, 2019)
Neuromyelitis optica spectrum disorder	In NMOSD, autoantibodies target aquaporin 4 on CNS cells, activating C1 and triggering the classical pathway and alternative pathway. This leads to MAC-mediated damage of astrocytes and neurons.	C5mAb (eculizumab)	(Pittock et al., 2019, 2023; Watanabe et al., 2024; Ringelstein et al., 2024; Kim et al., 2021)
Autoantibodies to the acetylcholine receptor positive generalized myasthenia gravis	In AChR + gMG, anti-AChR antibodies activate C1 at the neuromuscular junction, triggering the classical pathway and alternative pathway. This causes MAC-mediated damage to the neuromuscular junctions.	C5mAb (eculizumab and ravulizumab/ultomiris)	(Muppidi et al., 2019; Vu et al., 2023; Pane et al., 2024; Brandsema et al., 2024; Howard et al., 2017)
Atypical hemolytic uremic syndrome	In aHUS, loss-of-function mutations in complement regulator genes (CD46, CFH, CFI) and gain-of-function mutations (C3, CFB), along with autoantibodies against FH, increase activity in the alternative pathway. This leads to MAC-mediated kidney damage, platelet and endothelial activation (via C5aR1), and thrombotic microangiopathy.	C5mAb (eculizumab and ravulizumab/ultomiris)	(McKeage, 2019; Schönfelder et al., 2024; Busutti et al., 2024; Tanaka et al., 2021; Shahid and Qayyum, 2023; Tomazos et al., 2022; Dixon et al., 2024)
SARS-CoV-2 induced disease	In COVID-19, the SARS-CoV-2 spike protein directly activates the classical, lectin, and alternative pathways. It also triggers local C3 production and activation in type 2 pneumocytes. This leads to endothelial and epithelial cell damage (via C5aR1 and MAC) and recruits immune cells (via C3aR and C5aRs).	C5a mAb (vilobelimab/gohibic)	(van Amstel et al., 2024; Vlaar et al., 2022a, 2022b; Lim et al., 2022, 2023; Malone et al., 2024; McCarthy, 2023)
Anti-neutrophil cytoplasmic antibody associated vasculitis	In ANCAAV, autoantibodies target PR3 and MPO on PMNs, activating C1 and triggering the classical and alternative pathways. This leads to PMN activation, ROS production, NET formation, and endothelial cell activation (via C5aR1).	C5aR1 antagonist (avacopan/tavneous)	(Jayne et al., 2017, 2021; Geetha et al., 2024; Cortazar et al., 2023; van Leeuwen et al., 2025)

AChR (autoantibodies to the acetylcholine receptor), aHUS (atypical hemolytic uremic syndrome), AMD (age-related macular degeneration), ANCA (anti-neutrophil cytoplasmic antibody), ANCA-AV (ANCA-associated vasculitis), CAD (cold agglutinin disease), C (complement component), C3a (complement 3a), C3aR (C3a receptor), C5a (complement 5a), C5aR1 (C5a receptor 1), CF (complement factor), COVID-19 (SARS-CoV-2–induced disease), DAF/CD55 (decay-accelerating factor), gMG (generalized myasthenia gravis), iC3b (inactivated C3b), IgM (immunoglobulin M), MAC (membrane attack complex), MAC-IP/CD59 (MAC-inhibitory protein), MPO (myeloperoxidase), NET (neutrophil extracellular trap), NMOSD (neuromyelitis optica spectrum disorder), PNH (paroxysmal nocturnal hemoglobinuria), PMN (polymorphonuclear cells), PR3 (proteinase 3), RBCs (red blood cells), ROS (reactive oxygen species), SARS-CoV-2 (severe acute respiratory syndrome coronavirus 2).

To ensure a comprehensive and balanced synthesis, this review was informed by an extensive literature search across PubMed, Scopus, and Web of Science using combinations of the keywords, i.e., Adenylosuccinate lyase deficiency, SAICAr,purine metabolism, complement activation, neuroinflammation,cytokines, and inborn errors of metabolism. Articles were selected based on their relevance to the mechanistic link between metabolic dysfunction and immune activation, particularly those published within the last 10 years, though seminal older studies were also included. Key studies that informed our understanding of complement-cytokine interplay in neurodevelopmental and neurodegenerative contexts are cited throughout the paper.

This figure illustrates the classical pathway of complement activation, which is initiated when the C1 complex comprising C1q, C1r, and C1s binds to the Fc region of immunoglobulins (IgM or IgG) that are attached to antigens or released from damaged cells making IgG/IgM-specific immune complexes,(e.g., IgG-ICs/IgM-ICs) (a–b). Upon recognition of such immune complexes to their corresponding IgG receptor (FcγRs) or IgM receptor (FcμR), the serine proteases C1r and C1s cleave C4 and C2, forming the C4b2a complex, which is known as C3 convertase (C4b2a) (c-e). This C3 convertase cleaves C3 into C3a and C3b (f). C3a is a potent anaphylatoxin, causing cellular activation and promoting inflammation. C3b can then associate with the C4b2a to generate the C5 convertase (C4b2aC3b) (g), which in turn cleaves C5 into the pro-inflammatory fragment C5a and the MAC-initiating fragment C5b (h). The terminal pathway is initiated by C5b, which sequentially recruits C6, C7, C8, and multiple C9 molecules to form the membrane attack complex (MAC), ultimately resulting in pore formation and cell lysis (i).

This figure shows the lectin pathway of complement activation, which is initiated by the binding of mannose-binding lectin (MBL) or ficolins to specific carbohydrate motifs, such as mannose binding lectin receptor (MR) on the surface of pathogens or altered host cells (a). This binding activates MBL-associated serine proteases (MASP1 and MASP2), which cleave C4 and C2 to form the C3 convertase C4b2a (b–d). This convertase then cleaves C3 into C3a and C3b (e). C3a acts as chemoattractant and cellular activation agents, whereas like the classical pathway, C3b joins C4b2a to form the C5 convertase (f), which cleaves C5 into C5a and C5b (g). C5a functions as a powerful chemoattractant, guiding immune cells to sites of inflammation. In parallel, C5b triggers the terminal complement pathway by sequentially recruiting C6, C7, C8, and several C9 molecules to form the membrane attack complex (MAC). The MAC integrates into the membranes of target cells, creating pores that disrupt osmotic balance and lead to cell lysis, thereby facilitating the clearance of pathogens or damaged cells (h).

This figure illustrates the alternative pathway, which operates at a low basal level and is rapidly amplified in response to infection or tissue injury. The pathway is initiated by the spontaneous hydrolysis of C3 to C3(H2O) (a), which binds complement factor B (CFB) (b). Complement factor D (CFD) then cleaves CFB (c), generating the fluid-phase C3 convertase, C3(H2O)Bb (d). On microbial surfaces or damaged host tissues, C3b generated from any pathway binds to CFB and is again cleaved by CFD to form the surface-bound C3 convertase, C3bBb that causes the cleavage of C3 to C3a and C3b (e). This C3 convertase cleaves additional C3 molecules, creating an amplification loop that leads to the further cleavage of C3 to C3a and C3b. C3a interacts with its C3aR and activates the C3a-C3aR-mediated effector function, whereas an additional molecule of C3b reacts with C3bBb to form C5 convertase (C3bBbC3b) (f). The C5 convertase cleaves C5 into C5a and C5b (g). Acting as a strong signaling molecule, C5a attracts immune cells to areas of infection or injury. Meanwhile, C5b sets off the final phase of the complement cascade by binding to C6, C7, C8, and multiple C9 units, forming the membrane attack complex (MAC). This complex embeds into the surface of target cells, creating transmembrane pores that disrupt cellular homeostasis, resulting in cell rupture and the elimination of invading microbes or compromised host cells.

In Adenylosuccinate lyase deficiency disorder (ADSLDD), a genetic defect in the adenylosuccinate lyase (ADSL) enzyme leads to the cell accumulation of succinylaminoimidazole carboxamide ribotide (SAICAR)-induced dephosphorylated form, succinylaminoimidazole carboxamide riboside (SAICAr) in the bloodstream (a). This excessive buildup disrupts the synthesis of AICAR, a crucial metabolite essential for the regulation of complement factor B (CFB), which is key to initiating the alternative complement activation pathway (b). The resulting metabolic imbalance alters the dynamics of SAICAr, promoting its interaction with neighboring leukocytes, which ultimately leads to cellular injury (c). This cellular injury activates the alternative complement pathway, starting with the spontaneous hydrolysis of complement component C3 into C3(H2O) (d). This modified form of C3 binds to CFB, forming the enzyme complex C3(H2O) Bb (e). C3(H2O) Bb cleaves additional C3 into the fragments C3a and C3b (f). The generated C3b further binds to CFB, exposing a site for the enzymatically active complement factor D (CFD) to cleave CFB into its active forms, Ba and Bb, thus generating the potent C3 convertase (C3bBb) (g). This complex significantly amplifies complement activation, converting additional C3 into C3a and C3b (h). The C3bBb complex then engages further C3b to form the C5 convertase (C3bBbC3b), which cleaves C5 into C5a (i).

In Adenylosuccinate lyase deficiency disorder (ADSLDD), a genetic defect in the adenylosuccinate lyase (ADSL) enzyme leads to the accumulation of succinylaminoimidazole carboxamide ribotide (SAICAR)-induced dephosphorylated form, succinylaminoimidazole carboxamide riboside (SAICAr), in the bloodstream (a). This excessive buildup disrupts the synthesis of AICAR, a crucial metabolite essential for the regulation of complement factor B (CFB), which is key to initiating the alternative pathway of complement activation(b). The resulting metabolic imbalance alters the dynamics of SAICAr, promoting its interaction with neighboring leukocytes, which ultimately leads to cellular injury (c). The cellular injury activates the alternative complement pathway, starting with the hydrolysis of complement component C3 into C3(H2O) (d). This form of C3 binds to complement factor B (CFB), forming the enzyme complex C3(H2O)B (e), which is cleaved by complement factor D (CFD) to create the C3 convertase (C3bBb) (f), facilitating the conversion of C3 into C3a and C3b (e). The C3bBb complex then engages additional C3b to form the C5 convertase (C3bBbC3b), which cleaves C5 into C5a, as described in more detail in Fig. 1 g) The increased levels of complement components C3a and C5a subsequently engage their respective receptors (C3aR and C5aR1) on various immune cells, initiating a cascade of immune responses. h) This interaction enhances the expression of costimulatory molecules (e.g., CD40, CD80, CD86) on phagocytes, (e.g., monocyte, macrophage, and dendritic cells) and stimulatory markers (e.g., CD40L, CD69) on T cells, driving immune cell activation and resulting in a robust inflammatory response characterized by the release of pro-inflammatory cytokines such as IFNγ, IL1β, IL6, IL17, and TNFα. i) Elevated levels of these cytokines compromise the integrity of the blood-brain barrier (BBB), leading to its dysfunction. This dysfunction permits the infiltration of peripheral immune cells (e.g., monocytes, macrophages, and T cells) into the central nervous system (CNS). j) Within the CNS, infiltrating immune cells interact with neuronal cells, (e.g., astrocyte, oligodendrocyte, neuron, and microglial cells) exacerbating the inflammatory environment. k) This interaction between immune cells and neuronal cells amplifies cytokine production, resulting in cellular damage and the progression of neurodegeneration characteristic of ADSLDD.

## CRediT authorship contribution statement

**Albert Frank Magnusen:** Writing – review & editing. **Robert James Hopkin:** Writing – review & editing. **Charles Vorhees:** Writing – review & editing. **Elizabeth Wilson:** Writing – review & editing. **Molly Moehlman:** Data curation. **Barbara Hallinan:** Data curation. **Craig Erickson:** Writing – review & editing. **Melissa P. DelBello:** Writing – review & editing. **Luca Marsili:** Writing – review & editing, Data curation. **Nicole G. Coufal:** Writing – review & editing, Data curation. **Manoj Kumar Pandey:** Writing – review & editing, Writing – original draft, Visualization, Supervision, Software, Resources, Investigation, Data curation, Conceptualization.

## Funding

This study was conducted without external financial support.

## Declaration of competing interest

The authors declare that they have no known competing financial interests or personal relationships that could have appeared to influence the work reported in this paper.

## Data Availability

No data was used for the research described in the article.
